# Pharky, Phedro, Phractured, and StagePhright: complete genome sequences of four EK2 cluster Microbacterium phages

**DOI:** 10.1128/mra.00424-26

**Published:** 2026-07-17

**Authors:** Susan M. R. Gurney, Medha Gupta, Deeksha Reddy, Gloria Zacharias, Aleda S. Jomy, Carly J. Smith, Drexel University SEA-PHAGES annotators 2020, Drexel University SEA-PHAGES annotators 2021, Matthew T. McDonald, Brett M. Condon

**Affiliations:** 1 School of Biology, University of St Andrews, St Andrews, Scotland, United Kingdom; 2 Department of Biology, Drexel University 6527, Philadelphia, Pennsylvania, USA; 3 Bristol Myers Squibb, Princeton, New Jersey, USA; 4 Lewis Katz School of Medicine at Temple University 12314, Philadelphia, Pennsylvania, USA; 5 Charles River Laboratories, Horsham, Pennsylvania, USA; 6 Thomas Jefferson University 6559, Philadelphia, Pennsylvania, USA; 7 Department of Biomedical Sciences, Rocky Vista University–Montana College of Osteopathic Medicine 149990, Billings, Montana, USA; Queens College Department of Biology, Queens, New York, USA

**Keywords:** bacteriophage, EK cluster, *Microbacterium foliorum*

## Abstract

We report the complete genome sequence of four Microbacterium phages, Pharky, Phedro, Phractured, and StagePhright, isolated using the bacterial host *Microbacterium foliorum* from soil samples collected in Philadelphia. All four phages are genetically very similar and contain a gene of unknown function, which is uncommon in the EK2 cluster.

## ANNOUNCEMENT

Bacteriophages are an exciting area of research with clinical relevance. Phages have been successfully used in the clinical treatment of antibiotic-resistant bacterial infections in humans (1–6). These phages are isolated from environmental samples, including soil *Microbacterium* species, which are known to cause opportunistic pathogenic infections in humans (7–9).

**Fig 1:**
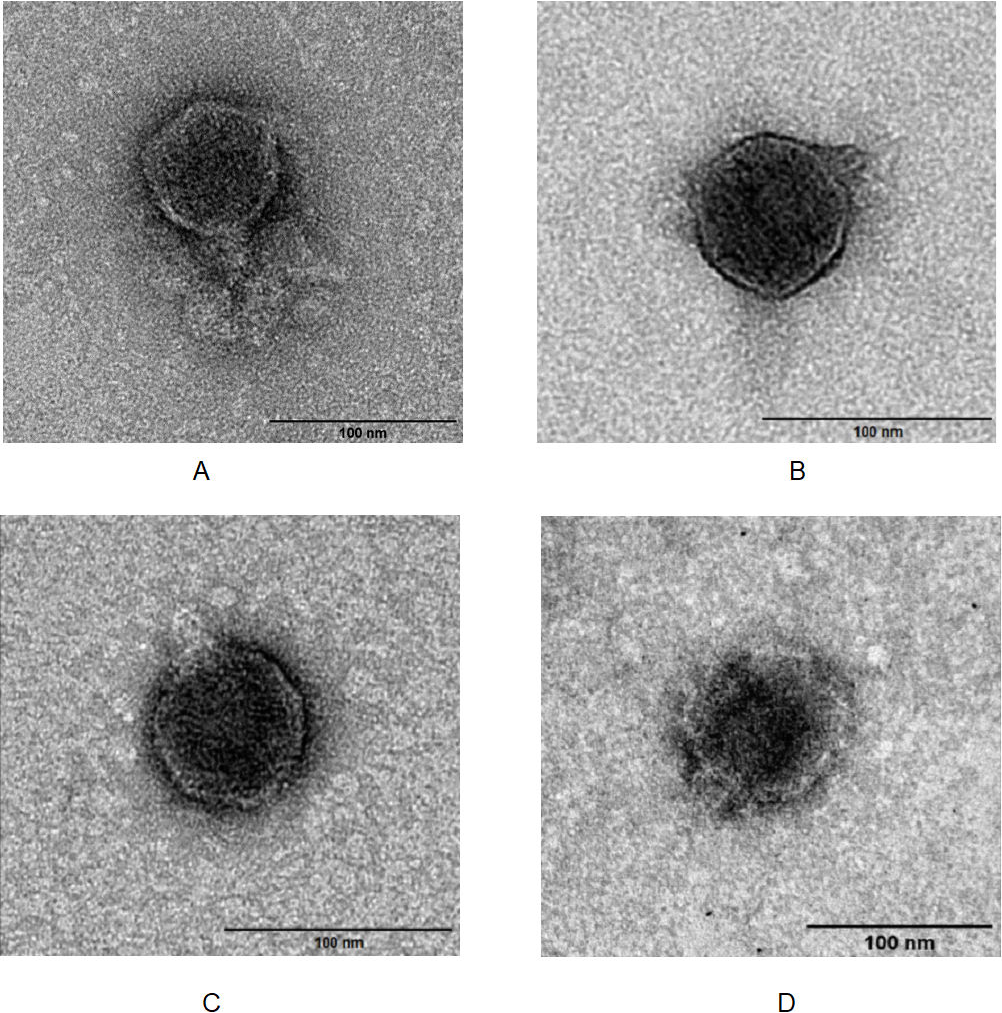
Transmission electron microscopy images of bacteriophage (**A**) Phractured, (**B**) Phedro, (**C**) Pharky, and (**D**) StagePhright. All have a podovirus morphology with an icosahedral capsid. The samples were stained with 1% uranyl acetate and imaged using a JEOL 2100F field-emission transmission electron microscope.

Here, we report the complete genome sequences of four EK2 cluster phages from the subfamily *Eekayvirinae*, the genus *Akonivirus*, and species *Akonivirus phedro*, isolated using *Microbacterium foliorum* (*M. foliorum*; NRRL B-23224) from soil samples collected in Philadelphia (39°57′21.6″N, 75°11′29.0″W). All four exhibit the expected Podoviridae morphology by transmission electron microscopy (Fig. 1) (10, 11). All contain a gene of unknown function (4789) found uncommonly in EK2 cluster phages.

Pharky, Phedro, Phractured, and StagePhright were isolated from soil using an enriched technique, as described previously (12). Briefly, 15 mL samples were added to 20 mL PyCa media and shaken for 2 h at 30°C. A 0.22 µm filter separated the media from soil particulates and was mixed with *M. foliorum* cultures and shaken for 2–5 days. The result was filtered and mixed with *M.* foliorum on PyCa plates. Individual bacteriophages were isolated from plaques, then purified through three rounds of plating, and finally prepared as a lysate from which genomic DNA was extracted using the Promega Wizard DNA Cleanup Kit (12). Sequencing libraries were prepared from genomic DNA with the NEB Ultra II FS Kit, and 48 libraries were multiplexed and sequenced at the Pittsburgh Bacteriophage Institute using the Illumina MiSeq platform (v3 reagents), providing at least 26-fold coverage with single-end 150 bp reads (Table 1). Raw reads were assembled with Newbler v2.9 using default parameters, resulting in a single contig. Each assembly was evaluated for completeness, accuracy, and genomic termini using Consed v29. Genome annotation was conducted utilizing DNA Master v5.23.3 (https://phagesdb.org/DNAMaster/), Glimmer v3.02 (13), GeneMark v2.5p (14), Starterator (https://phages.wustl.edu/starterator/), Phamerator (using Actino_draft database v578) (15), PhagesDB BLAST (using Actinobacteriophage database) (https://phagesdb.org/blast/), National Center for Biotechnology Information (NCBI) BLASTP (using NCBI non-redundant database) (16), the NCBI CDD (17), HHPRED (using the PDB_mmCIF70, Pfam- v.36, NCBI Conserved Domains databases) (18), Aragorn v1.2.38 (19), tRNAscan-SE v2.0 (20), and PECAAN (https://discover.kbrinsgd.org). Default settings were used for all software. All four phages were assigned to cluster EK2 based on gene content similarity of at least 35% to phages in the Actinobacteriophage database, phagesdb (https://phagesdb.org) (21, 22).

**TABLE 1:** New EK2 phages

Phage name	Subcluster	GenBank accession number	SRA accession number	Length (bp)	G + C (%)	Protein coding genes (#)	Number of reads	Genome sequencing coverage	Genomic termini
Phractured	EK2	MT310889	SRX31360681	5,442	59.7	55	127,031	235	Circularly permuted
Phedro	EK2	MT310890	SRX32748209	5,442	59.7	55	125,502	266	Circularly permuted
Pharky	EK2	MT310891	SRX32748208	5,442	59.7	55	112,588	192	Circularly permuted
StagePhright	EK2	MW862991	SRX31360683	5,442	59.7	55	70,356	39	Circularly permuted

An NCBI BLAST comparison of these four novel EK2 phages shows extremely high similarity across the full genome. Compared to Pharky, Phedro has 100% coverage with 99.98% sequence identity; StagePhright has 100% coverage with 99.97% sequence identity; and Phractured has 100% coverage with 99.96% sequence identity. Other phages in the cluster show more diversity; for example, Fede (MT684587) has 68% coverage and only 78.27% sequence identity compared to Pharky.

Phamerator analysis shows all four of these novel EK2 phages contain a small gene of unknown function (4789) that is only present in six other EK2 phages. In contrast, all phages described here have a minor tail protein (86976) that is common to 70% of EK2 phages.

## Data Availability

Accession numbers for our sequences are provided in Table 1 under BioProject PRJNA488469.
